# Segmentation of LiDAR point cloud data in urban areas using adaptive neighborhood selection technique

**DOI:** 10.1371/journal.pone.0307138

**Published:** 2024-07-18

**Authors:** Debobrata Chakraborty, Emon Kumar Dey

**Affiliations:** Institute of Information Technology, University of Dhaka, Dhaka, Bangladesh; Universidade Federal de Uberlandia, BRAZIL

## Abstract

Semantic segmentation of urban areas using Light Detection and Ranging (LiDAR) point cloud data is challenging due to the complexity, outliers, and heterogeneous nature of the input point cloud data. The machine learning-based methods for segmenting point clouds suffer from the imprecise computation of the training feature values. The most important factor that influences how precisely the feature values are computed is the neighborhood chosen by each point. This research addresses this issue and proposes a suitable adaptive neighborhood selection approach for individual points by completely considering the complex and heterogeneous nature of the input LiDAR point cloud data. The proposed approach is evaluated on high-density mobile and low-density aerial LiDAR point cloud datasets using the Random Forest machine learning classifier. In the context of performance evaluation, the proposed approach confirms the competitive performance over the state-of-the-art approaches. The computed accuracy and F1-score for the high-density Toronto and low-density Vaihingen datasets are greater than 91% and 82%, respectively.

## Introduction

Three-dimensional (3D) Light Detection and Ranging (LiDAR) point cloud data segmentation is a prominent area of research in remote sensing, photogrammetry, and computer vision. Due to the rapid development of technology, it is now possible to obtain LiDAR point cloud data using mobile laser scanning (MLS), terrestrial laser scanning (TLS), and aerial laser scanning (ALS) [1]. Those technologies can extract LiDAR point cloud data from a complex urban environment and the acquired data have been used in various 3D urban scene analysis applications, including building extraction [2–4], road identification [5], power line identification [6], vegetation cover analysis [7], and urban scene segmentation [8, 9]. The individual point in aerial LiDAR point cloud data contains Cartesian coordinates (*X*, *Y*, *Z*), where *X*, *Y*, and *Z* represent each point’s latitude, longitude, and height, respectively. In addition to these coordinates, it may also contain additional properties, such as RGB color information. The segmentation of individual points is quite challenging due to outliers, partial loss, and uneven density in the captured LiDAR point cloud data [10].

The existing approaches of LiDAR point cloud data segmentation can be categorized into geometric rule-based [7, 11], machine learning-based [1, 12–14], and deep learning-based approaches [15, 16, 61]. The machine learning-based approaches usually consist of three essential steps: selecting a neighborhood for each 3D point, extracting feature values for all points based on the identified neighborhoods, and training and testing by using a supervised classifier based on the extracted features [14].

As a key step in point cloud segmentation, traditional neighborhood recovery methods, including *k*-Nearest Neighbor (*k*-NN) [17], spherical neighborhood [18], and cylindrical neighborhood [19], can select neighboring points based on a fixed size of *k*, radius (*r*), or height (*h*) value for all points in a dataset. In these methods, the size of neighborhoods must be arbitrarily determined without having any knowledge of the point cloud dataset. Selecting appropriate neighborhood points is essential as it enables the estimation of local surface orientations by extracting the geometric features of an object. Geometric features can be calculated based on different combinations of eigenvalues, which are extracted by analyzing the 3D covariance matrix of a 3D point and its neighborhood. Therefore, selecting the wrong neighborhood can lead to significant errors in eigenvalue calculation and may cause inaccurate extracted features.

Urban scenes contain objects with distinct geometric shapes, such as buildings, trees, and roads. In the case of a fixed *k*-NN approach, it may select all neighboring points from a small area due to the higher point density, or it may include neighbors from outliers, different planes, or different objects. In the case of fixed spherical or cylindrical neighborhoods, selecting the neighborhood in a missing point area may also be difficult. To determine neighborhood scale parameters, including height, radius, or *k* values, a comprehensive understanding of the urban scene is necessary. This issue can be addressed by employing an adaptive neighborhood selection strategy.

Among the state-of-the-art adaptive neighborhood selection approaches, there are several methods including entropy-based [1], curvature-based [14, 20], and omnivariance-based [21] techniques. Omnivariance-based, and entropy-based methods calculate local geometric features using various *k* neighborhood sizes. The neighborhood with the minimum omnivariance or entropy is considered the optimal selected neighborhood. The omnivariance-based neighborhood selection method proposed by Günen [21] does not utilize geometric features to divide the input point cloud data into different regions for neighborhood selection. This can lead to inappropriate neighborhood selection and feature extraction by choosing neighboring points from different regions or objects that do not correspond to the intended object point. The curvature-based neighborhood selection method divides the input point cloud data into regular and scatter regions based on curvature features. Following this division, the entropy-based neighborhood is applied separately to the regular and scatter regions. To further enhance this method, Xue et al. [14] select neighborhood points based on distance and normal angle within a spherical radius around each point in the scatter region. Here, the regular region includes points from building roofs, facades, and roadways, whereas the scatter region encompasses points from object edges, corners, and vegetation [14]. However, the authors did not consider instances where the neighborhood point of a specific object is selected from a different object class or a different surface plane within these two regions. Thus, clustering the input point cloud into multiple regions or understanding the classes beforehand enables the selection of neighboring points with similar attributes or geometric characteristics.

To address these issues, regular and scattered regions need to be further divided into additional regions to select the neighborhood appropriately. In addition, an adaptive neighborhood value selection approach based on each point’s geometric region is required. This approach should select neighboring points that have comparable geometric features and ensure that the points are chosen from similar geometric regions.

The particular contributions presented in this research are as follows:

- Based on correlations between points and several geometric properties, including curvature, verticality, and omnivariance, the input LiDAR point cloud dataset is divided into four distinct regions.
- For each of the distinct regions, appropriate adaptive neighborhood selection techniques are used to solve the problems of the existing fixed-scale neighborhood. A simplicial complex-based neighborhood selection approach is introduced in the highly dispersed region of the urban area point cloud.
- Appropriate geometric features are computed for each region using the adaptively selected neighborhood techniques to enhance the overall effectiveness of the urban point cloud segmentation.

The rest of this paper is organized as follows: the Literature Review section provides an overview of current state-of-the-art methodologies. Following that, the proposed neighborhood selection technique for various regions is described in the Methodology section. The Experiments section elaborates on the detailed experiments conducted, while the Results subsection discusses the findings derived from these experiments.

## Literature review

The main objective of this research is to find suitable neighbors for each point in the input point cloud to calculate the accurate feature value for segmenting the LiDAR point cloud data. This section first discusses relevant studies on existing LiDAR point cloud segmentation and then discusses the existing neighborhood selection techniques.

### Point cloud segmentation

Different geometric rule-based, machine learning-based, and deep learning-based approaches exist in the literature to analyze the aerial, terrestrial, and mobile LiDAR point cloud data that contain different objects. In the rule-based approach, a set of geometric rules is established for every object based on its geometry and elevation aspects [7]. For example, Vega et al. [22] introduced the rule-based PTrees technique, which is a multi-scale dynamic point cloud segmentation approach for recovering forest trees from LiDAR point clouds by employing raw elevation values (*Z*) and height computation (*H* = *Z*—ground elevation). Awrangjeb et al. [23] presented a rule-based segmentation approach for building roof plane extraction where the raw LiDAR data was divided into ground and non-ground points. Then, non-ground points were segmented to extract the building planar roof. Dey et al. [24] used a robust plane fitting method based on M-estimator SAmple Consensus (MSAC) to classify buildings from the input LiDAR point cloud data. The main problem with the rule-based approach is selecting the appropriate threshold value for the chosen geometric parameters (e.g., angle, curvature, and normal). Setting a threshold globally is challenging due to the heterogeneous nature of LiDAR point cloud data.

In the machine learning-based approach, the extracted features from the input point cloud data are fed into a machine learning classifier to train the system, and then the system can make predictions for new or unseen point cloud test data [21, 25]. Different machine-learning classifiers, including Random Forest [26], Support Vector Machine (SVM) [27], AdaBoost [28], Decision Tree [21], Linear Discriminant Analysis [21], Bayesian Discriminant Analysis [29], LightGBM [60], and Few-Shot Learning [62] have been used by different authors in the literature for the semantic segmentation of LiDAR point cloud data. Xue et al. [14] conducted a comparative analysis of point cloud segmentation using various machine learning classifiers to work on adaptive neighborhood selection. In the same year, Jiang et al. [30] investigated multispectral airborne LiDAR point cloud classification with different machine learning classifiers, observing higher accuracy with Random Forest. The main issue with the machine learning-based approach is selecting suitable features and selecting optimal neighborhood selection to increase the effectiveness of classifiers [14, 20, 21, 30].

Recent research has emphasized the use of deep learning algorithms to improve current methods and accuracy [15, 16, 31–33]. PointNet, a deep learning model, has proven to be beneficial for point cloud classification and segmentation, but it overlooks the relationship between points and local neighborhoods [34]. To consider the local neighborhood during deep learning classification, A-CNN [35], 3P-RNN [36], and DGCNN [37], PointCNN [31] have been proposed, which can extract geometric features and consider the neighborhood. Han et al. [61] presented a deep learning model that includes components for spatial downsampling, feature abstraction, and addressing class imbalance for semantic segmentation of urban scenes. To capture the relationship between points and their neighbors, Nong et al. proposed a PointNet++ method incorporating elevation information interpolation to improve object discrimination in ALS point classification tasks [32]. Along with that, a multilayer perception using a neighborhood selection was also utilized by Amakhchan et al. [38] and Fayez et al. [16] for urban scene classification.

In all instances, selecting the appropriate neighborhood is crucial for point cloud deep learning and machine learning models, and has a direct impact on their performance [33]. It influences the computation of various geometric properties and the efficacy of deep learning and machine learning models. The following subsection details the existing literature on neighborhood selection techniques.

### Neighborhood selection

Traditional neighborhood recovery methods such as *k*-NN, spherical neighborhood, and cylindrical neighborhood can calculate features for machine learning classifiers based on a fixed-size neighborhood for each point in the input point cloud data. The *k*-NN method is defined as the *k* number of points that are nearest to a selected point according to the Euclidean distance. Here, the number of neighbors is determined according to a fixed number of *k* for all points in a dataset [39]. Weinmann et al. [40] used a fixed *k*-NN to calculate the features for semantic segmentation of the LiDAR point cloud. Chen et al. [41] used *k*-NN with a fixed value of *k* to segment boundary points. The spherical neighborhood method of a selected point is defined as all points included in a sphere with a predefined radius (*r*) around the origin selected point. The *r* is the main parameter of this neighborhood method [18]. Li et al. [42] employed a spherical neighborhood selection with radius values of 0.2m, 0.5m, and 0.8m for urban scene classification. Mallet et al. [27] used a fixed spherical neighborhood based on the point density of the dataset. Another method developed for selecting the neighborhood size is the cylindrical neighborhood [43]. This method determines the neighborhood based on volumetric calculations. In cylindrical neighborhood selection, the radius (*r*) and height (*h*) of the cylinder are the two main factors that affect the success of the method. The authors of the literature [28, 44, 45] utilized various adaptations of cylindrical neighborhood methods to segment diverse urban objects from the input LiDAR point cloud data.

However, fixed-size neighborhoods may not accurately capture the geometric features of all objects in the input LiDAR point cloud data of urban scenes [46]. To avoid the limitation of fixed-size neighborhoods, several authors proposed adaptive approaches to neighborhood selection [20, 47–49]. Weinmann et al. [1] used a Shannon entropy-based adaptive method to select the *k* value for each point individually in the input point cloud data. They computed eigenentropy to select neighboring points and extracted features to segment urban scenes based on that neighborhood selection. Eigenentropy means the order or disorder of points, as well as the amount of uncertainty associated with these eigenvalues. They considered different values of *k* and chose the *k* value, which calculates the minimum entropy for each point in a point cloud dataset. He et al. [20] proposed a curvature-based adaptive neighborhood selection method. Based on a calculated threshold of curvature value, the author divided the input point cloud data into scatter and regular regions. Then, they used a *k*-minimal entropy-based neighborhood selection for scatter regions and a *r*-minimal entropy-based neighborhood for regular regions. Günen [21] presented a neighborhood recovery method based on the omnivariance property. The author defined a lower bound and an upper bound of *k*. Then, he calculated the omnivariance of a point and selected the *k* value with the lowest omnivariance value. Peng et al. [50] employed a controlled search radius experiment within a specific range to find the best-sized neighborhood for their used datasets. Dey et al. [46] proposed a method to determine the *k* value for *k*-NN in order to find an optimal neighborhood initially. The proposed method uses the *k*-NN algorithm to select a minimal number of neighboring points (k = 3) for a specific point *P*_*i*_. A best-fit 3D line is then constructed using these neighboring points, and the standard deviation of the calculated distances is compared to a distance threshold. A distance threshold is computed by using point density, which is equal to the distance between two neighboring points. If the standard deviation is below the threshold, the value of *k* is increased iteratively to find the minimal neighborhood for *P*_*i*_, ensuring an accurate plane’s normal estimation in LiDAR point cloud data. Xue et al. [14] proposed an adaptive neighborhood selection method based on the method proposed by He et al. [20]. The authors observed that corner points have higher curvature values and worked specifically with the scatter set of points. A large spherical radius for neighborhood selection was employed in the scatter set of points, and then excess points within the sphere were filtered out based on a selected threshold, which was calculated from the distance and normal angle between the points. In 2023, Jiang et al. [30] proposed maximum entropy-based neighborhood selection for multispectral airborne LiDAR point cloud segmentation. In the same year, Sevgen et al. [60] converted irregular points into a regular format using radius search with grid sampling before implementing the machine learning classifier.

In all cases of point cloud segmentation, implementing an adaptive neighborhood selection for feature computation is more effective than utilizing a fixed-size neighborhood selection [14, 20, 21, 30]. During neighborhood selection, points of an entire urban scene can be divided into regions based on several geometrical properties. For each region, an appropriate adaptive method can be proposed for selecting the neighborhood, which can enable the selection of points with similar geometrical properties as well as belonging to the same class, thereby minimizing outlier issues. The next section describes the proposed method to select an appropriate neighborhood for individual points to facilitate an effective segmentation of the input point cloud data.

## Methodology

**Fig 1:**
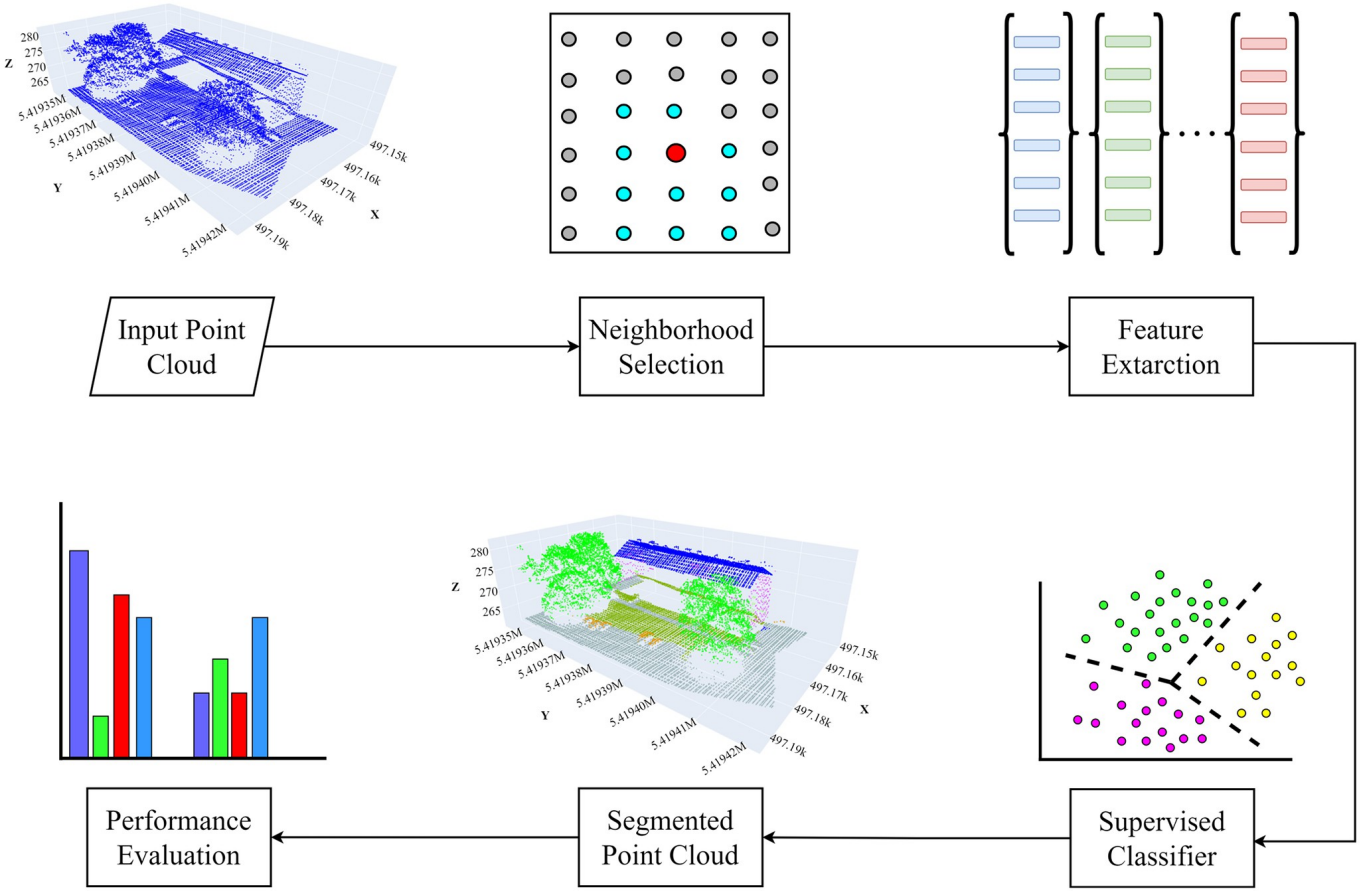
The general architecture of the point cloud data segmentation.

The general architecture of LiDAR point cloud data segmentation using machine learning is illustrated in Fig 1. The selection of neighborhoods serves as the basis for feature extraction. Considering the geometric variation in the input point cloud data, the next subsection presents the method for selecting an appropriate neighborhood approach. The feature extraction subsection provides an overview of the selected features for this study, and subsequently discusses the supervised classifier model utilized in this study.

### Proposed neighborhood selection method

The literature review describes the importance of selecting appropriate neighborhoods in urban areas for point cloud analysis. However, these studies did not take into account the diverse geometric regions within the urban area point cloud dataset during neighborhood selection. Although the curvature-based [14, 20] neighborhood selection technique divides the input point cloud data into regular and scatter regions, to select neighborhood points more accurately from similar objects, similar planes, or similar geometric features, we use the verticality feature to divide the regular region into planar and vertical regions. Additionally, we divide the scatter region into low and high omnivariance areas using the omnivariance feature. As urban point clouds can be categorized into distinct regions based on geometric features, by dividing the input point cloud into four regions, suitable neighborhoods are chosen. To choose an appropriate neighborhood, the initial step of the proposed method involves categorizing the urban point clouds into four different regions, as follows:

- Planar region,
- Vertical region,
- Low omnivariance region, and
- High omnivariance region.

**Fig 2:**
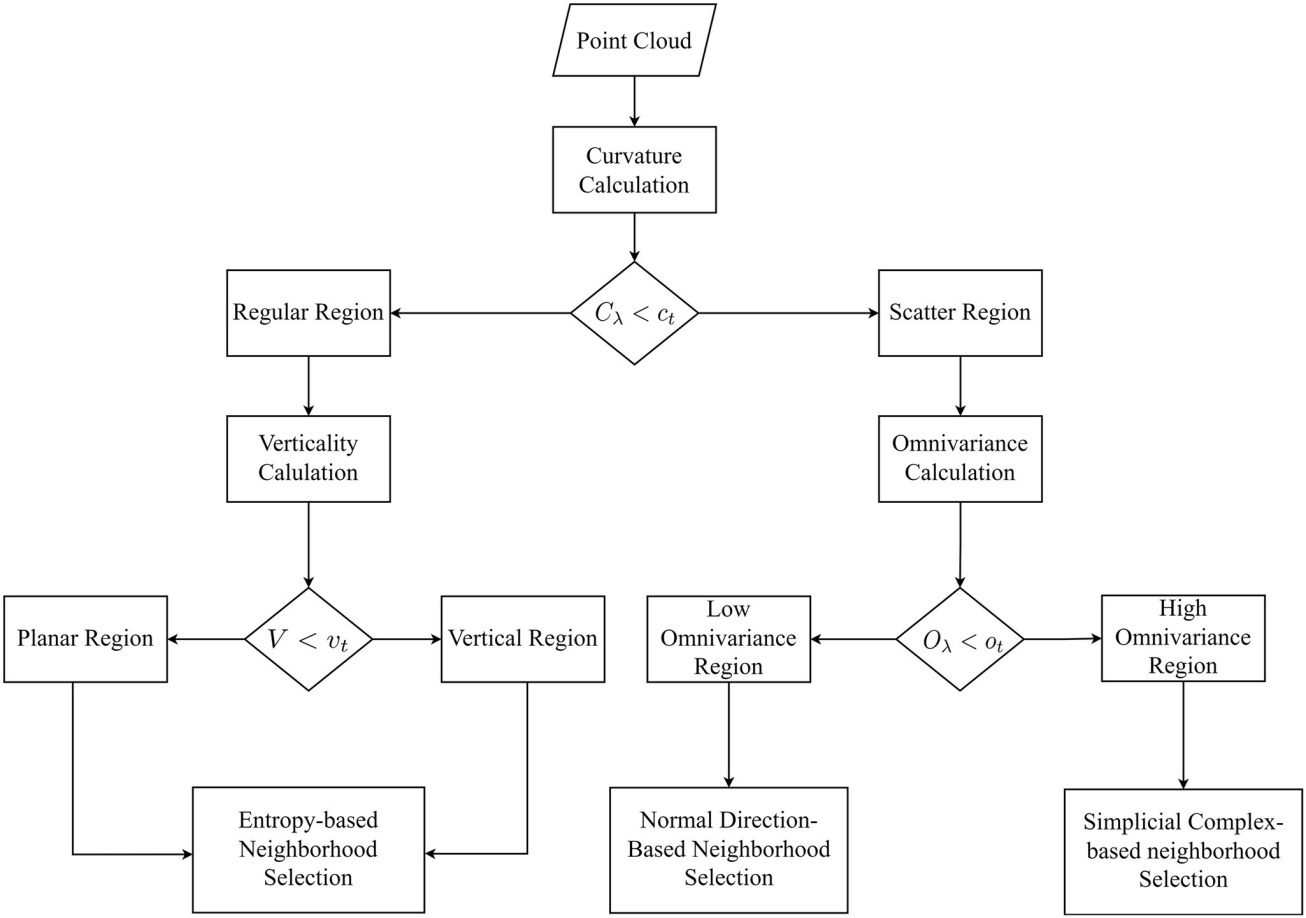
Framework of our proposed neighborhood selection method.

A distinct neighborhood selection process is employed based on the categories of the regions. For the planar and vertical regions, an entropy-based method is utilized. A neighborhood selection method based on the direction of the normal is used in the low omnivariance region, and a simplicial complex-based method is used for neighborhood selection in the high omnivariance region. This approach ensures that throughout the neighborhood selection process, points from similar geometric regions are chosen as neighbors. The overall framework is illustrated in Fig 2.

First, based on the curvature geometric property of each point, the input point cloud data is separated into two regions using the approach proposed by He et al. [20]. The curvature (*C*_λ_) is calculated using Eq (1) and based on a curvature threshold (*c*_*t*_) the input data is separated into regular (*P*_*r*_) and scattered regions (*P*_*s*_) using Eq (2). Here, the eigenvalues λ_1_, λ_2_, and λ_3_ are extracted from a 3D covariance matrix. The curvature threshold value (*c*_*t*_) is selected following the method of He et al. [20] and Xue et al. [14].

$$\begin{array}{c} C_{\lambda }=\frac{\lambda_{3}}{\lambda_{1}+\lambda_{2}+\lambda_{3}} \end{array}$$ (1)

$$\begin{array}{c} P_{c}=\{\begin{array}{cc} P_{r}, & \text{if}\;C_{\lambda }<c_{t}\\ P_{s}, & \text{if}\;C_{\lambda }\geq c_{t} \end{array} \end{array}$$ (2)

The points in the regular region are further separated into planar and vertical regions based on the verticality (*V*) property of the input point cloud. The verticality (*V*) of any point *P*_*i*_ is calculated using the normal vector (*n*_*x*_, *n*_*y*_, *n*_*z*_) [51], as shown in Eq (3) where *n*_*z*_ represents the third component of the normal vector of a point. The calculation of the normal vector is performed utilizing the weighted PCA method as mentioned in [46]. Following the approach mentioned by Xue et al. [14] for curvature threshold, the verticality threshold (*v*_*t*_) is determined. Lastly, Eq (4) is used to separate the points into planar and vertical regions.

$$\begin{array}{c} V=1-n_{z} \end{array}$$ (3)

$$\begin{array}{c} P_{i}=\{\begin{array}{cc} P_{planar}, & \text{if}\;V<v_{t}\\ P_{vertical}, & \text{if}\;V\geq v_{t} \end{array} \end{array}$$ (4)

The geometric characteristic omnivariance *O*_λ_ of a point *P*_*i*_ can be calculated using Eq (5). To separate the points into low and high omnivariance regions, we use the Eq (6) where the threshold *o*_*t*_ is also retrieved based on the method utilized for curvature threshold by Xue et al. [14]. The distinctive neighborhood selection method is then applied to each of the four regions in the input LiDAR point cloud data to extract the feature value for segmentation purposes.

$$\begin{array}{c} O_{\lambda }=3\sqrt{\lambda_{1}+\lambda_{2}+\lambda_{3}} \end{array}$$ (5)

$$\begin{array}{c} P_{i}=\{\begin{array}{cc} P_{low}, & \text{if}\;O_{\lambda }<o_{t}\\ P_{high}, & \text{if}\;O_{\lambda }\geq o_{t} \end{array} \end{array}$$ (6)

**Fig 3:**
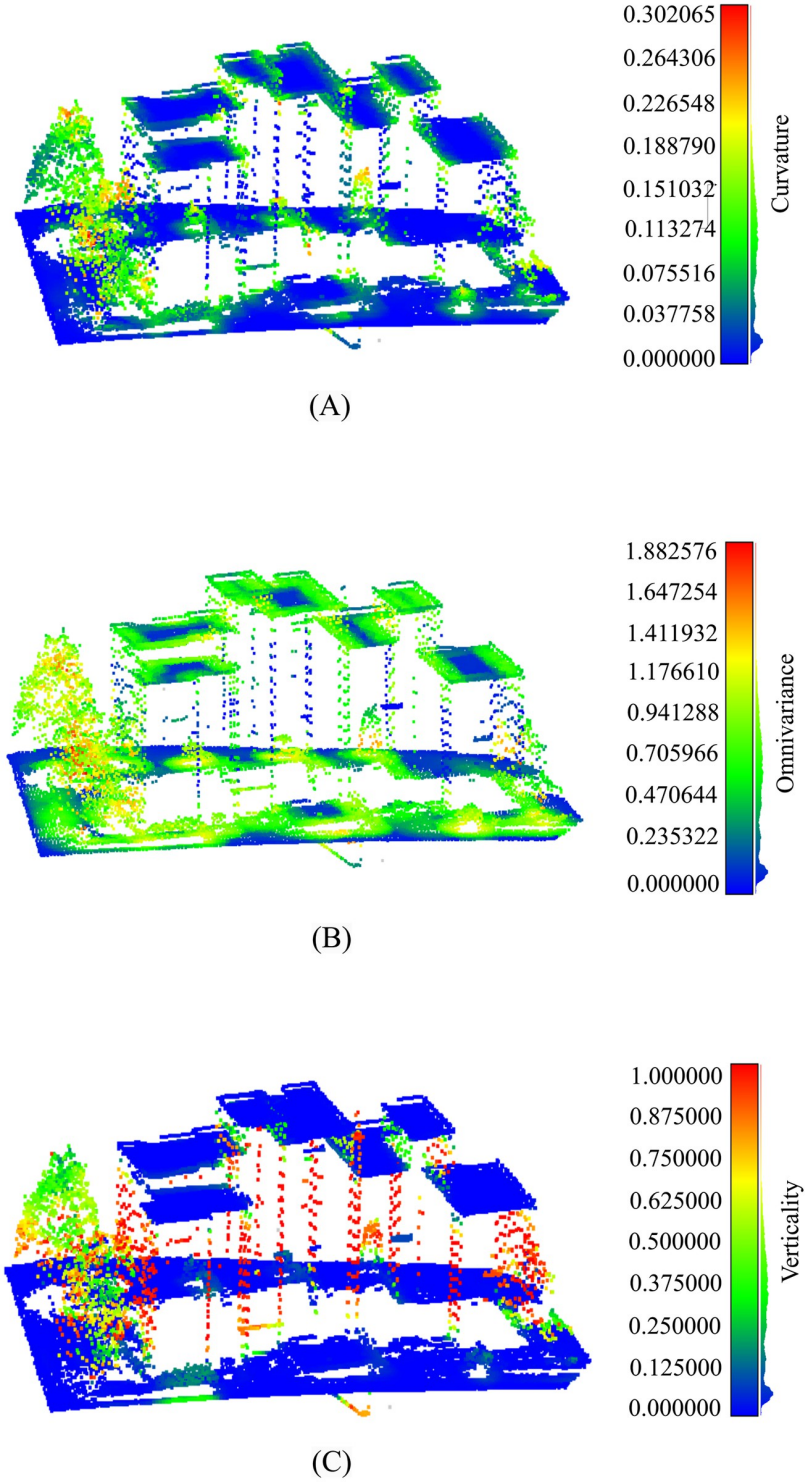
Visualization of features. (A) Curvature, (B) Omnivariance, and (C) Verticallity features of individual points from a portion of an urban area.

Fig 3 demonstrates the values of curvature, omnivariance, and verticality of individual points from a small portion of an urban area.

#### Entropy-based neighborhood

For the regular region of the input point cloud data, we choose the entropy-based neighborhood selection approach. The entropy-based method begins by calculating the eigenentropy or Shannon entropy of a point *P*_*i*_ using Eq (7), where *e*_*i*_ = λ_*i*_/Σ_λ_ with *i* ∈ 1, 2, 3. Various values of *k* nearest neighbors are then considered for any point *P*_*i*_, and the *k* value with the lowest entropy is chosen as the most appropriate neighboring point for *P*_*i*_ [52].

$$\begin{array}{c} E_{\lambda }=-e_{1}\;\text{ln}\left(e_{1}\right)-e_{2}\;\text{ln}\left(e_{2}\right)-e_{3}\;\text{ln}\left(e_{3}\right) \end{array}$$ (7)

Points on the building roof, ground surface, and horizontal surface are referred to as planar points, whereas any vertically elevated points, such as the building facade or electrical pole, are referred to as vertical points. However, there might be a situation where points from a building roof or a ground surface are included as neighborhoods of a building facade or other vertical surfaces during the process of identifying neighboring points. Additionally, the entropy-based neighborhood selection method may also select neighborhood points from different regions. This could lead to errors in the calculation of the value of the accurate feature [46]. Our method avoids this problem, as we previously divided the points into vertical and horizontal planar regions. The verticality feature is used to divide the regular points into two sets to reduce these particular types of problems. As a result, points carrying similar geometric features will be selected as neighborhood points. The entropy-based neighborhood selection method is applied separately for each region to determine the neighborhood with the least amount of disorder.

#### Normal direction-based neighborhood selection

In LiDAR point cloud data, the scatter points mostly cover vegetation and object corners or edges [14]. However, there might be a situation where building edges and trees are too close to each other. For example, during the neighborhood selection of a building edge point, it may select neighboring points from scattered tree points or other objects near the building, or vice versa. To acquire an appropriate neighborhood, the scatter region is divided into two regions based on omnivariance, as described earlier. Omnivariance is a characteristic based on eigenvalues that describes how points spread or disperse in different directions [21]. The points of trees and shrubs are more widely dispersed in diverse directions. Thus, vegetation has a larger omnivariance value than the corners or edges of an object.

**Fig 4:**
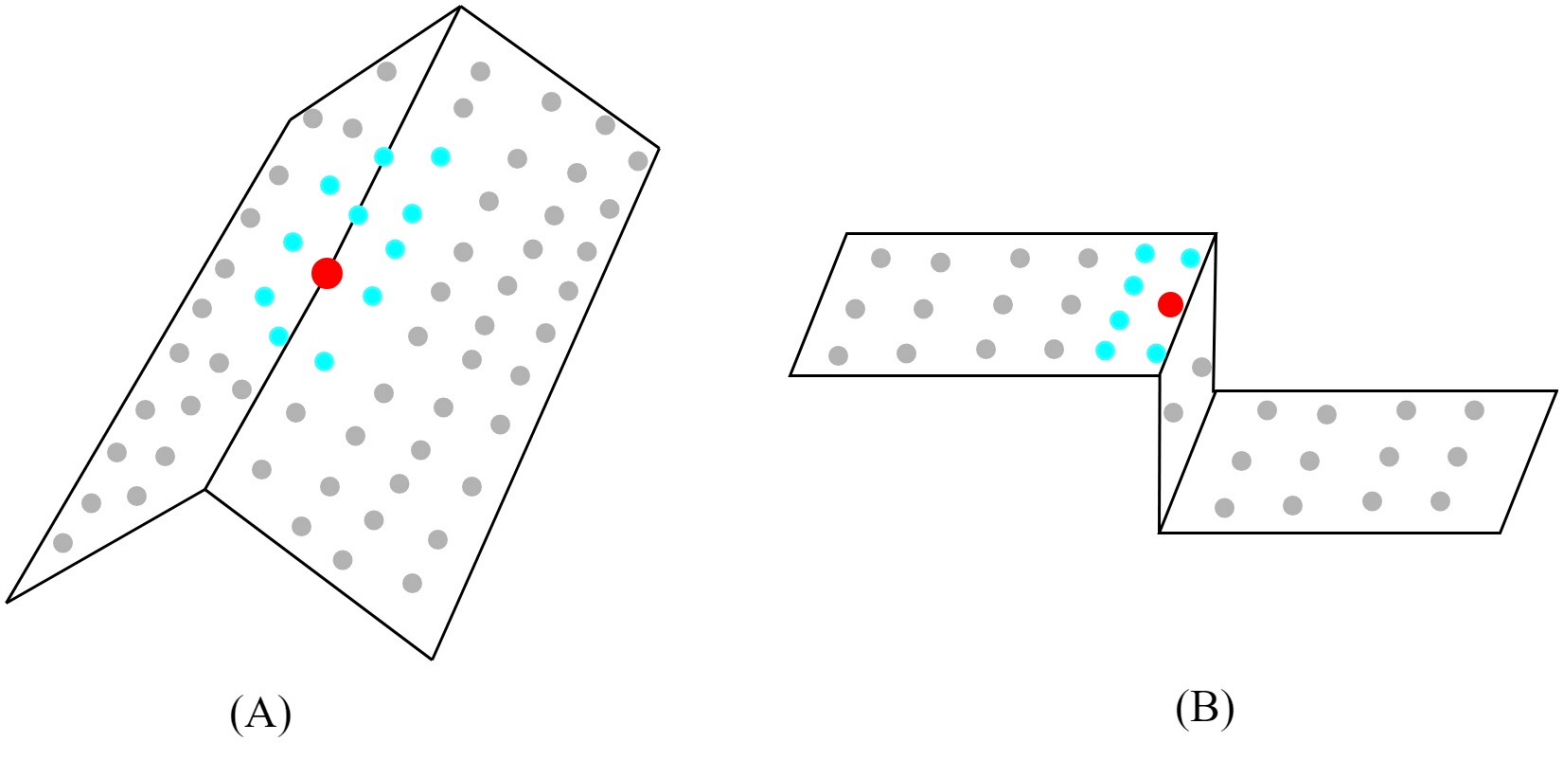
An example of adding points to the neighborhood in the low omnivariance region. (A) Fold points, (B) Edge points between planes. Here, red points represent Point *P*_*i*_, and cyan points indicate the selected neighborhood.

As the low omnivariance region (*P*_*low*_) mostly covers building edges and corners, variable-size neighborhoods for each point *P*_*i*_ are selected adaptively based on the direction of the normal vector of the nearby points. Initially, the *k* value for the nearest neighbor is selected using the method proposed by Dey et al. [46]. The method starts with a minimum value for *k* and iteratively increases the value of *k* until the standard deviation of the selected neighboring points satisfies a threshold value *θ* computed based on the average point density of the input point cloud. After determining the neighborhood points *S*_*p*_ of a point *P*_*i*_, the direction of the normal of each neighboring point is computed. The normal angle is taken into account to determine the proposed appropriate neighborhood. Then, the average of the normal angles (*η*_*p*_) of points in *S*_*p*_ is calculated. If the normal angle of any points in *S*_*p*_ is less than *η*_*p*_, then we consider that point as a neighborhood of *P*_*i*_ in the low omnivariance regions of the input point cloud data. This ensures the selection of neighboring points within the fold points of an object, as shown in Fig 4A. Furthermore, if any point *P*_*i*_ is on a horizontal or vertical plane edge, the neighboring points are selected from the same plane as *P*_*i*_, as shown in Fig 4B.

#### Simplicial complex-based neighborhood selection

**Fig 5:**
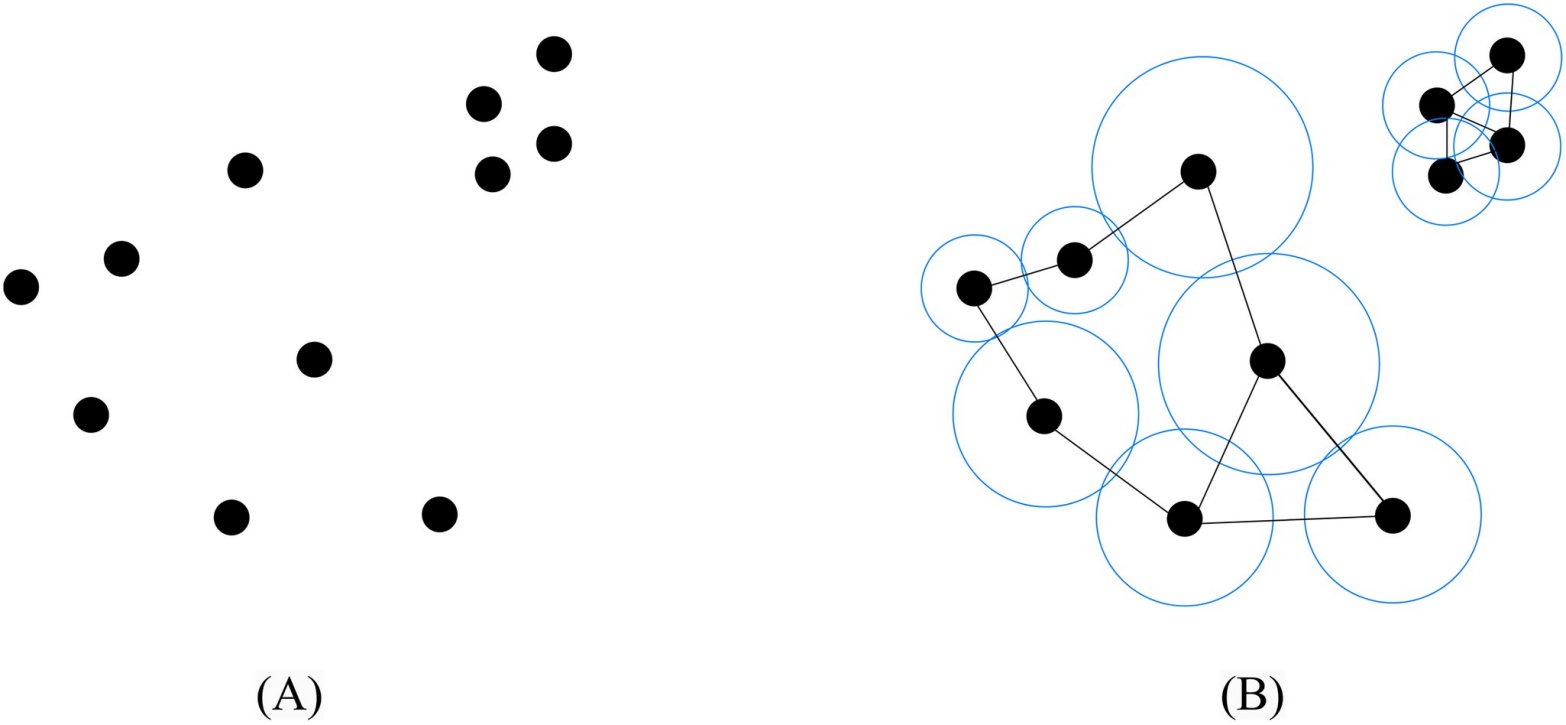
Simplicial complex-based neighborhood selection. (A) Point Cloud Data, (B) Choosing the neighborhoods.

The high omnivariance region encompasses the majority of rougher surfaces, such as vegetation points, shrub areas, and outlier points [53]. The selection of neighboring points from the same class or region is expected. To select appropriate neighboring points for any point *P*_*i*_ in the high omnivariance region, we utilized a method based on the simplicial complex-based neighborhood selection technique. The concept of a simplicial complex, presented by Zomorodian et al. [54], is a key aspect of the Persistent Homology method [55], which is used in topological data analysis. The simplicial complex-based method facilitates the clustering of similar points in a group, which can be considered neighbors of each other. The 3D covariance matrix can be calculated to extract features for each cluster when there are at least three different neighboring points for each point *P*_*i*_ [56]. The process began with an initial radius value considering the density of the input point cloud and then gradually expanded until any point in the cluster failed to reach at least two additional points. Every point in a cluster is connected to its neighbors by edges. Fig 5A demonstrates an example of scatter point cloud data with high omnivariance, and Fig 5B illustrates how the neighborhood is connected based on the simplicial complex-based neighborhood selection technique.

This approach can mitigate the impact of the isolated outlier points group, as the outlier points will be connected within themselves and will not attract points from any other object. Compared to entropy-based neighborhood selection, this approach will immediately locate the neighborhood for all the scattered points with high omnivariance value. The entropy-based approach is not suitable for high omnivariance regions because it requires different values of *k* to find the best one, which is time-consuming, and sometimes *k* could attract outlier points, leading to inaccurate feature value calculation for the scattered high omnivariance region.

### Feature extraction

Features of point clouds encompassing various characteristics of the point cloud data are extracted to train machine learning or deep learning models for classification and segmentation [14, 57]. The feature values are calculated after the local neighborhood size is determined for each point. Various features can be extracted from point clouds. For instance, eigenvalue-based features [1, 14, 20, 21, 46] and elevation features have also been explored in several studies [14]. Additionally, radiometric features are derived from multispectral LiDAR point cloud data [30]. This study employs a range of eigenvalue- and elevation-based features to enhance the effectiveness of 3D point cloud segmentation.

#### Eigenvalue-based features

The 3 × 3 covariance matrix is used to determine the eigenvalues λ_1_, λ_2_, and λ_3_ for a 3D point *P*_*i*_, where λ_1_ ≥ λ_2_ ≥ λ_3_ ≥ 0 [21]. In this study, nine eigenvalue-based features such as the sum of eigenvalues (Σ_λ_), omnivariance (*O*_λ_), eigenentropy (*E*_λ_), anisotropy (*A*_λ_), planarity (*P*_λ_), linearity (*L*_λ_), curvature (*C*_λ_), sphericity (*S*_λ_), and mean curvature (*M*_λ_) are used, respectively. Σ_λ_ characterizes the overall variance of a point along with its neighboring points, and *O*_λ_ aids in determining the dispersion of points across various directions [21]. *E*_λ_ determines the ordered or disordered points [1]. *P*_λ_, and *L*_λ_ are employed to assess the degree of planarity and linearity of a point [46]. The value of *S*_λ_ represents areas with high spheres. Anisotropy *A*_λ_ is a characteristic that describes the existence of sharp edges or corners [21]. The curvature *C*_λ_, also known as surface variation, represents the geometric attribute indicating the change in the shape of local surfaces [14, 20, 58]. Table 1 shows the selected eigenvalue-based features used in this paper.

**Table 1:** Eigenvalue-based features.

Features Name	Equation
Sum of Eigenvalues	Σ_λ_ = λ_1_ + λ_2_ + λ_3_
Eigenentropy	$E_{\lambda }=-\sum_{i=1}^{3}\lambda_{i}\text{ln}\left(\lambda_{i}\right)$
Omnivariance	$O_{\lambda }=3\sqrt{\lambda_{1}+\lambda_{2}+\lambda_{3}}$
Anisotropy	$A_{\lambda }=\frac{\lambda_{1}-\lambda_{3}}{\lambda_{1}}$
Planarity	$P_{\lambda }=\frac{\lambda_{2}-\lambda_{3}}{\lambda_{1}}$
Linearity	$L_{\lambda }=\frac{\lambda_{1}-\lambda_{2}}{\lambda_{1}}$
Curvature	$C_{\lambda }=\frac{\lambda_{3}}{\lambda_{1}+\lambda_{2}+\lambda_{3}}$
Sphericity	$S_{\lambda }=\frac{\lambda_{3}}{\lambda_{1}}$
Mean curvature	$M_{\lambda }=\frac{\lambda_{1}+\lambda_{2}}{2}$

#### Elevation features

Along with the eigenvalue-based features, we have used five additional elevation-based features, as shown in Table 2. *H*_*ave*_ represents the average elevation value of all neighboring points, including the selected point *P*_*i*_ in the point cloud [14]. *H*_*i*_ represents the elevation of the *i*-th individual point *P*_*i*_, and *N* denotes the total number of points within the neighborhood. *H*_*d*_ indicates elevation difference, where *H*_*highest*_ is the highest *Z* coordinate value and *H*_*lowest*_ is the lowest *Z* coordinate value within the neighborhood of *P*_*i*_. *H*_*a*_ represents the difference between the elevation of the current point and the point with the highest elevation value, denoted as *H*_*highest*_, in its neighborhood. *H*_*b*_, on the other hand, denotes the difference between the elevation of the current point and the point with the lowest elevation value, denoted as *H*_*lowest*_, in its neighborhood.

**Table 2:** Elevation-based features.

Features Name	Equation
Height Information	*H*_*i*_
Elevation Variance	$H_{v}=\frac{1}{N}\sum_{i=1}^{N}{\left(H_{i}-H_{\text{ave}}\right)}^{2}$
Elevation Difference	*H*_*d*_ = *H*_highest_ − *H*_lowest_
Height Above	*H*_*a*_ = *H*_*i*_ − *H*_highest_
Height Below	*H*_*b*_ = *H*_*i*_ − *H*_lowest_

### Supervised classifier

The training set of point cloud data is represented by {(*X*_*i*=1…*m*_, *L*_*n*_)}, where *X*_*i*_ represents features, and here *m* number of features are used for each point *P*_*i*_, while *L*_*n*_ signifies the corresponding semantic label of point *P*_*i*_. Using the selected different features mentioned in the previous subsection, a supervised machine learning classifier is used to train and test the dataset. The test dataset is used to assign predicted semantic labels to each data point, and these labels are then used to evaluate the performance of the classifier. The Random Forest (RF) classifier is selected as a representative of conventional classifiers because of its feasible and comprehensive adoption in the field of point cloud segmentation [57]. The Random Forest algorithm constructs multiple decision trees during training. Each tree can predict a class for every individual point.

## Experiments

In this section, the datasets used for the experiments are described first. Following the dataset description, the details of the experimental setup and the evaluation metrics used in this research are discussed. Finally, the experimental results are presented alongside the necessary findings of this study.

### Dataset description

**Fig 6:**
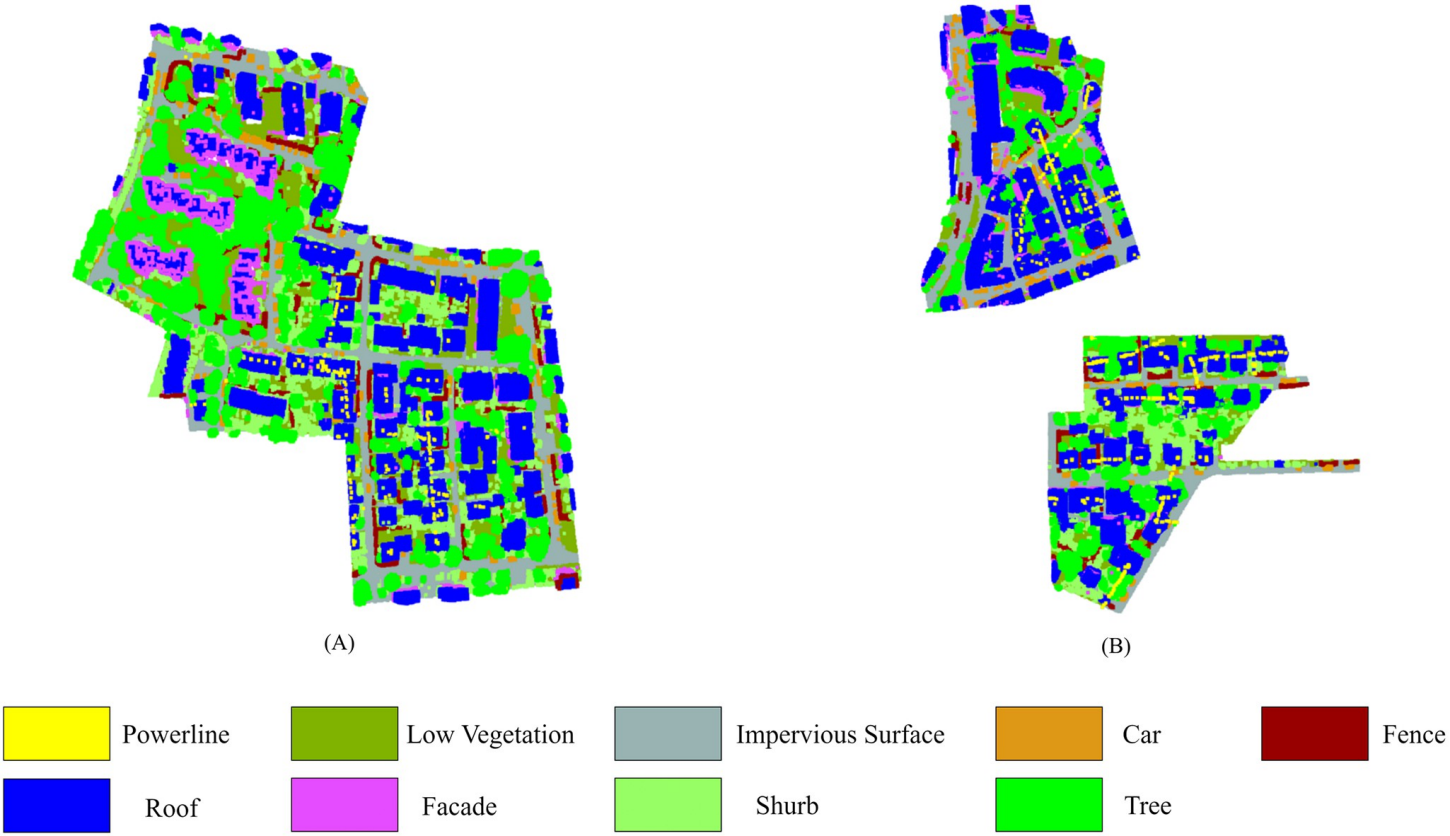
The LiDAR point cloud of ISPRS Vaihingen 3D benchmark dataset. (A) Site 1 for training, (B) Site 2 and 3 for testing. The legend at the bottom indicates the segmentation labels rendered in colors.

The aerial LiDAR point cloud dataset from the Vaihingen (VH) area, provided by the International Society for Photogrammetry and Remote Sensing (ISPRS) benchmark, and a mobile LiDAR point cloud dataset of the Toronto area are used to validate the performance of this research. An aerial Leica ALS50 scanner was used to collect the Vaihingen dataset from an altitude of 500 m at an angle of 45° [48]. The point density of the dataset ranges from 4 to 8 points/m^2^, and the points in this data set are not evenly distributed [32]. The dataset contains a total of nine semantic categories, including cars, facades, fences, impervious surfaces, low vegetation, roofs, power lines, shrubs, and trees. It contains three area sites with a total of 411,722 points in the two test sites and 753,876 points in one training site. Fig 6 demonstrates the LiDAR point cloud data of the training and test sites of the Vaihingen area.

**Fig 7:**
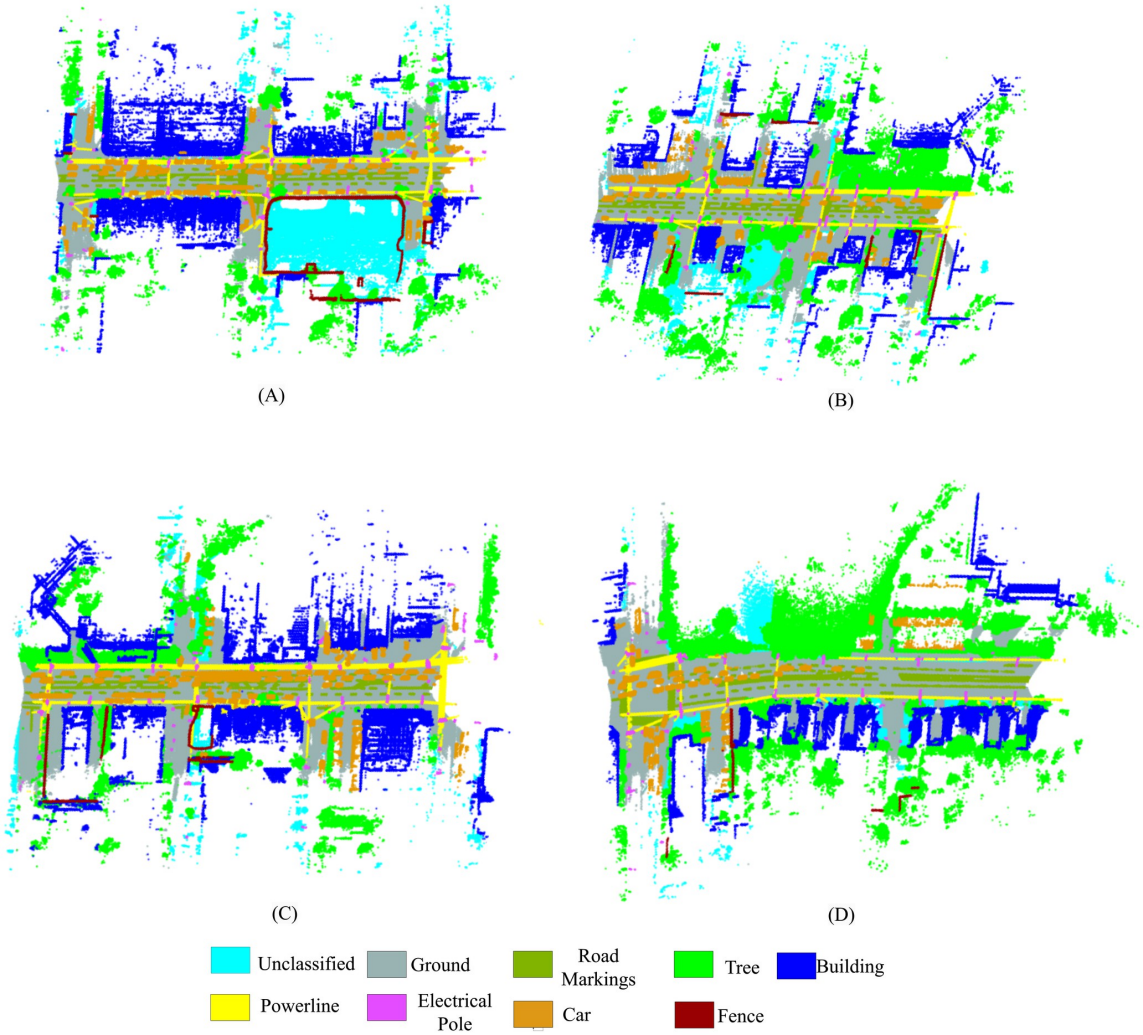
The Toronto-3D benchmark LiDAR point cloud dataset. (A) Site L001 for training, (B) Site L002 for training, (C) Site L003 for training, (D) Site L004 for testing. The legend at the bottom indicates the segmentation labels rendered in colors.

The Toronto-3D dataset is from Avenue Road in Toronto, Canada, which contains 6.7 million points with a high point density of approximately 1000 points/m^2^ on road surfaces [59]. The dataset contains a total of nine classes, including ground, road markings, trees, buildings, powerlines, electrical poles, cars, fences, and unclassified. The Toronto dataset contains four different parts denoted as L001, L002, L003, and L004. This study uses the L001, L002, and L003 areas for training and the L004 area for testing. Fig 7 illustrates the LiDAR point cloud data of the Toronto area used for our experimental purposes. Tables 3 and 4 show the distribution of the total number of points in the training and test sets for each class in the Vaihingen area of the ISPRS benchmark datasets and the Toronto area of the Toronto-3D LiDAR dataset, respectively.

**Table 3:** Number of points per category in training and test sets of the Vaihingen area of ISPRS benchmark dataset.

Class	Train Set	Test Set
Powerline	546	583
Low vegetation	180,850	198,491
Impervious surfaces	193,723	100,013
Car	4614	2231
Fence	12,070	2176
Roof	152,045	63,766
Facade	27,250	5732
Shrub	47,605	11,569
Tree	135,173	27,161
**Total**	**753,876**	**411,722**

**Table 4:** Number of points (thousand) per category in training and test sets of the Toronto-3D dataset.

Class	Train Set	Test Set
Unclassified Points	2511	582
Ground	38,118	3,738
Road marking	1,520	281
Tree	5,258	1,310
Building	18,575	525
Powerline	626	37
Electrical Pole	826	71
Car	3,732	200
Fence	407	4
**Total**	**71,573**	**6,748**

### Experimental setup

The experiment is implemented using the Python programming language. The Open3D library is used to visualize the 3D input point cloud data. Principal Component Analysis (PCA) is used as a base to estimate the significant eigenvalue-based geometric features of the points. Scikit-learn Python library is used to implement the Random Forest machine learning classifier model. The most appropriate class for a point *P*_*i*_ is chosen by a majority vote in the Random Forest classifier, which is fed the earlier described selected computed features for every point as input. Consequently, this chosen class becomes the final prediction for each point in the input point cloud data. This experiment is carried out using a set-up comprising an 11th-generation Intel Core i5 processor with a clock speed of 2.70 GHz, 16 GB of DDR4 RAM, a 4 GB NVIDIA RTX 3050Ti graphics processing unit, and the Windows 11 operating system.

### Evaluation measures

A confusion matrix for actual and predicted classes is formed using conventional parameters True Positive Rate (TP) (the ratio of the number of correctly classified points to the total number of points in this class), True Negative Rate (TN) (the ratio of the number of incorrectly detected points to the total points in that class), False Positive Rate (FP) (the ratio of the number of points in a class identified as a wrong class to the total points in a class), and False Negative Rate (FN) (the ratio of the number of correct points in a class identified as another class to the total correct point in that class). Based on these parameters, four commonly used evaluation metrics, such as Accuracy, Recall, Precision, and F1-score are used for evaluation using the following equations:

$$\begin{array}{c} \text{Accuracy}=\frac{\text{TP}+\text{TN}}{\text{TP}+\text{TN}+\text{FP}+\text{FN}} \end{array}$$ (8)

$$\begin{array}{c} \text{Recall}=\frac{\text{TP}}{\text{TP}+\text{FP}} \end{array}$$ (9)

$$\begin{array}{c} \text{Precision}=\frac{\text{TP}}{\text{TP}+\text{FN}} \end{array}$$ (10)

$$\begin{array}{c} F1-\text{score}=\frac{2\times \text{Precision}\times \text{Recall}}{\text{Precision}+\text{Recall}} \end{array}$$ (11)

### Results

This section presents the extensive experimental results. The impact of the calculated feature values, using the proposed neighborhood selection approach described in the methodology section, for the different regions is compared with state-of-the-art techniques.

**Fig 8:**
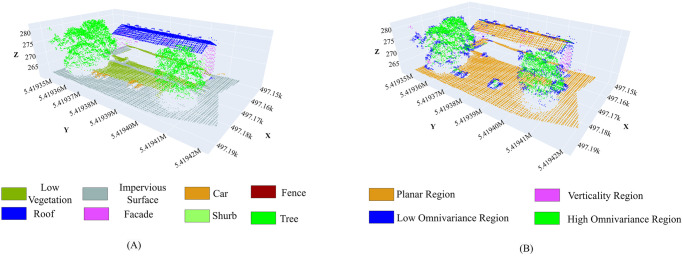
A portion of Vaihingen point cloud data. (A) Labeled ground truth, (B) Four distinct regions of the portion.

A portion of the LiDAR point cloud data from the Vaihingen area is used for demonstration purposes in Fig 8. Here, Fig 8A shows the ground truth of the selected area where each class is distinctly colored for clarity. Fig 8B depicts the initially separated four regions, including planar, vertical, low omnivariance, and high omnivariance, based on the proposed approach.

**Fig 9:**
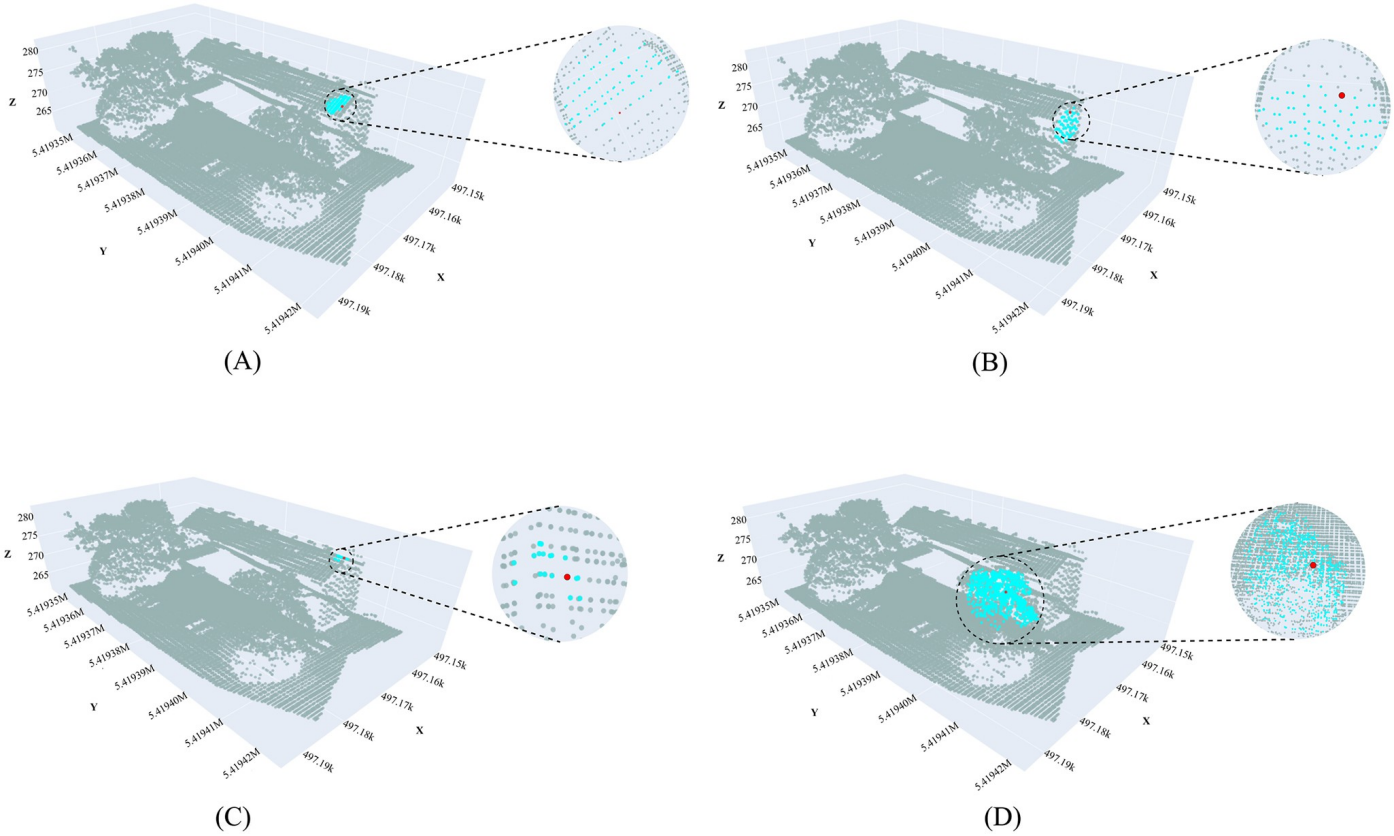
Neighborhood selection from distinct region. (A) Planar Region, (B) Vertical Region, (C) Low Omnivariance Region, and (D) High Omnivariance Region. The red point indicates any point *P*_*i*_, and the cyan color indicates the corresponding selected neighborhood.

Fig 9 demonstrates the outcomes of the proposed neighborhood selection methods for any point *P*_*i*_ based on its associated region. Fig 9A represents the selected neighborhood from a planar region using the entropy-based neighborhood selection approach. Fig 9B depicts the selected neighboring points in a vertical region using the same approach. Fig 9C shows the selected neighborhood in a low omnivariance region using the approach based on the direction of the normal. Finally, Fig 9D illustrates the selected neighborhood in a high omnivariance region based on the simplicial complex neighborhood selection method. In all of the cases, the point *P*_*i*_ is represented by the red color, while the selected neighborhood points are highlighted in cyan color.

The highlighted red point (*P*_*i*_) in Fig 9A is a point from the building roof, and it is clear that the neighboring points are also selected from the same roof plane. Fig 9B depicts that the neighboring points are only selected from the building’s vertical facade area. The points of the tree are also vertical; since they are initially divided into separate regions based on the value of curvature, those points are not taken into consideration for the vertical facade area.

The low omnivariance region mainly contains the points of the object’s edges and corners as shown in Fig 8B. Thus, a point *P*_*i*_ from rooftop fold points in Fig 9C selects the neighboring points only from the low omnivariance fold area of the building based on the angle of the normal. Additionally, the high omnivariance regions, encompassing the points of vegetation, including trees and shrubs, are depicted in Fig 8B. In this instance, *P*_*i*_ is chosen from a tree identified as a high omnivariance region. Here, multiple neighborhood points are selected, each derived from vegetation utilizing the simplicial complex-based neighborhood selection method.

Table 5 represents a quantitative performance evaluation of the proposed neighborhood selection methods on the Vaihingen test dataset. The proposed approach is compared with the state-of-the-art neighborhood selection methods of Nong et al. [32], Xue et al. [14], He et al. [20], Günen [21], Weinmann et al. [52]. We have also experimented with the point cloud test data using some fixed *k* nearest neighbors method using the features described in the eigenvalue-based and elevation feature section. The table shows that our proposed method outperforms all current neighborhood approaches in terms of evaluation metrics.

**Table 5:** The accuracy, precision values, recall values, and F1-score values according to different neighborhood selection methods of the Vaihingen dataset.

Method	Accuracy	Precision	Recall	F1-score
Proposed	0.913	0.874	0.784	0.82
Nong et al. [32]	0.857	0.727	0.778	0.751
Xue et al. [14]	0.795	0.729	0.573	0.642
He et al. [20]	0.909	0.856	0.76	0.8
Günen [21]	0.877	.574	0.689	0.63
Weinmann et al. [52]	0.864	0.781	0.555	0.639
*k*=100	0.809	0.765	0.656	0.697
*k*=50	0.801	0.783	0.645	0.694

**Fig 10:**
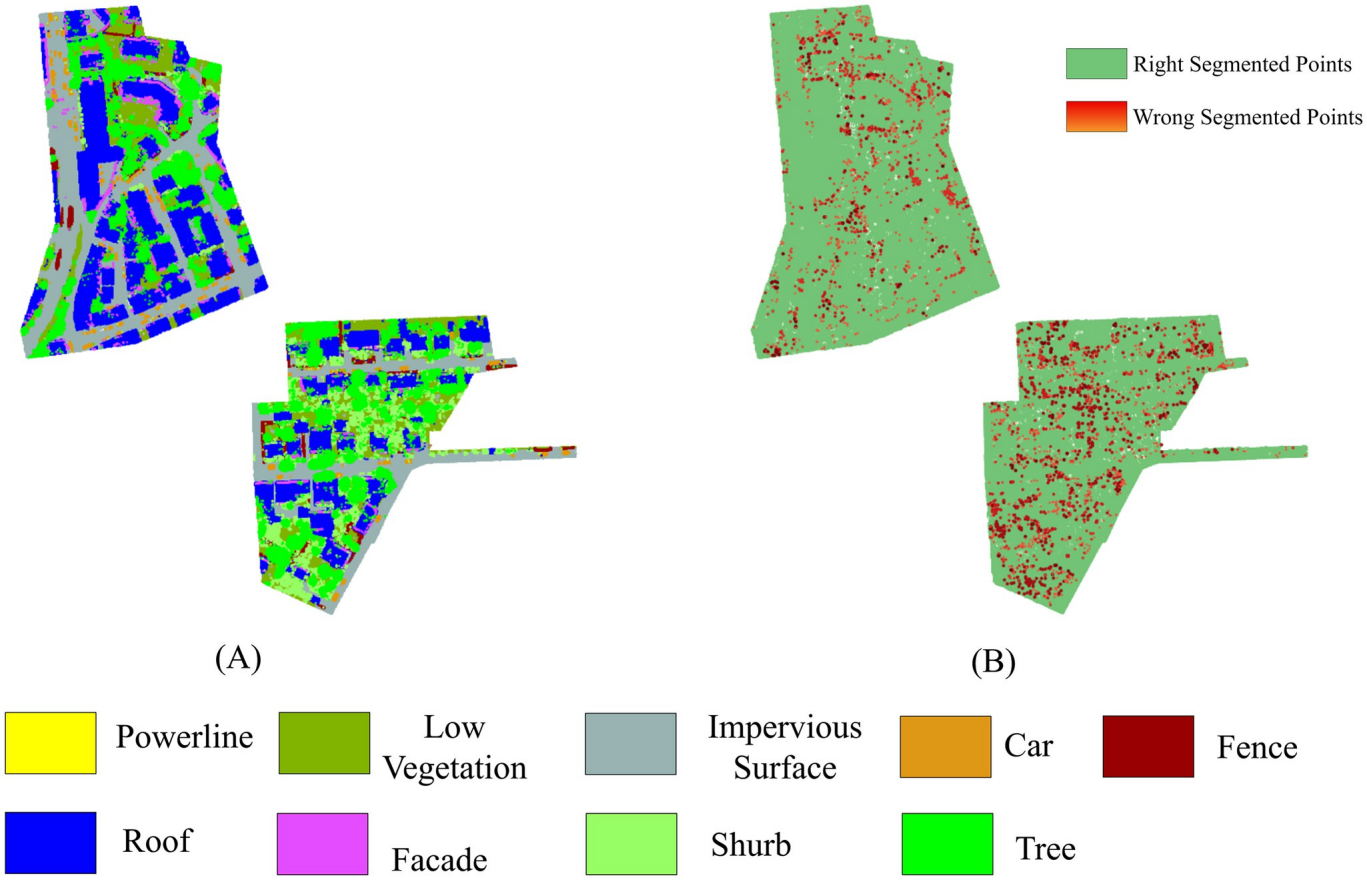
(A) Prediction map and (B) error map of the proposed method on the Vaihingen dataset.

**Fig 11:**
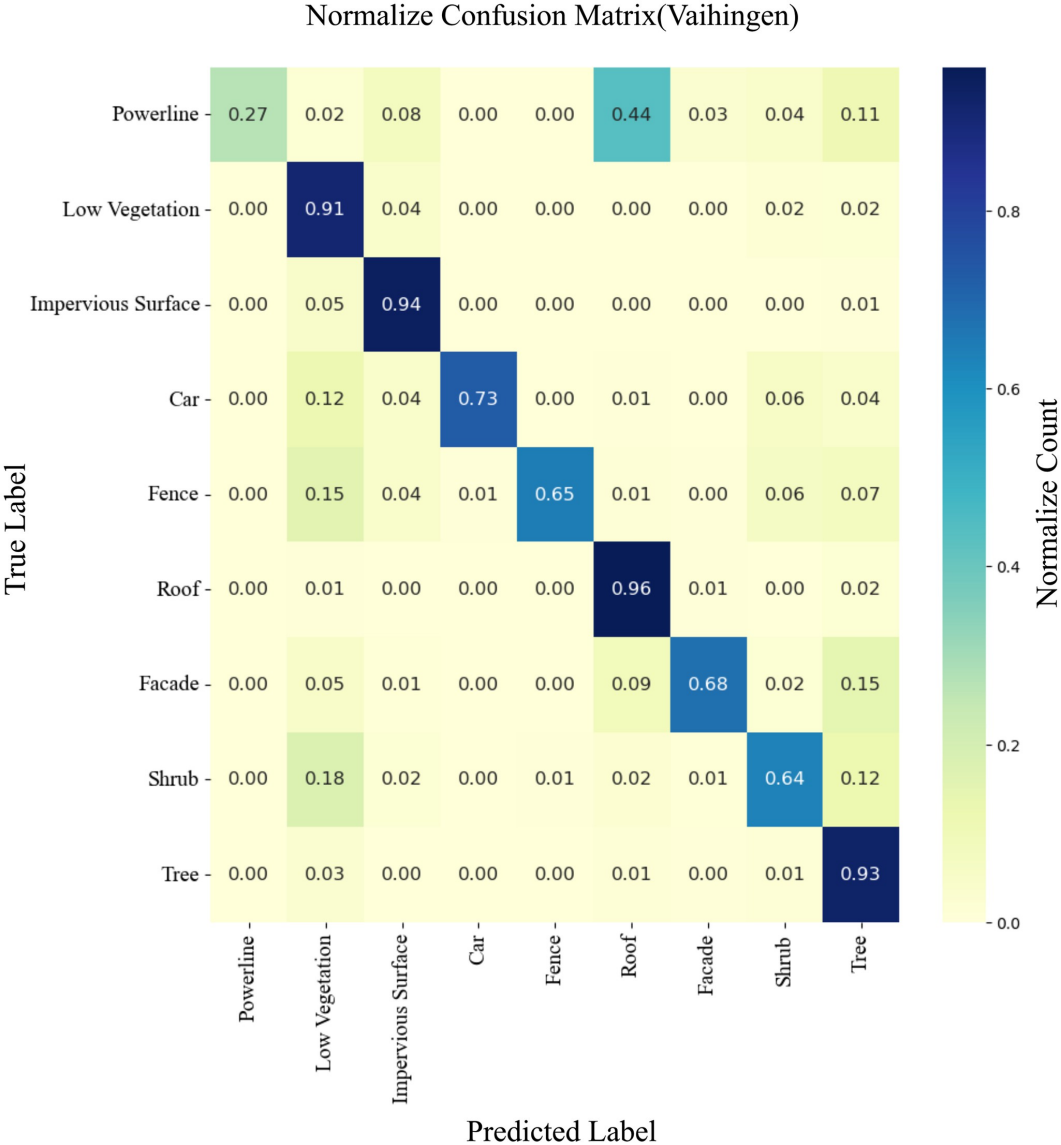
Confusion matrix for the machine learning classifier using proposed neighborhood approach on Vaihingen test dataset.

To demonstrate the performance of the machine learning classifier visually, LiDAR point cloud data of urban areas are segmented using distinct colors. Fig 10A illustrates that the majority of the points in the test scenes are accurately segmented. The misidentified points are shown with an error map in Fig 10B. Subsequently, the confusion matrix is represented in Fig 11, which shows that the true positive rate is satisfactory, particularly for identifying points corresponding to low vegetation, impervious surface, roof, and high omnivariance regions, which included the trees. However, some significant shrub points are misidentified as low vegetation and trees. Furthermore, a notable observation in the confusion matrix is the misidentification of most powerline points as roof points. This is because there are comparatively very few powerline points in the dataset for training. The machine learning classifier often misidentified the elevated nature of powerline points as roof points.

**Fig 12:**
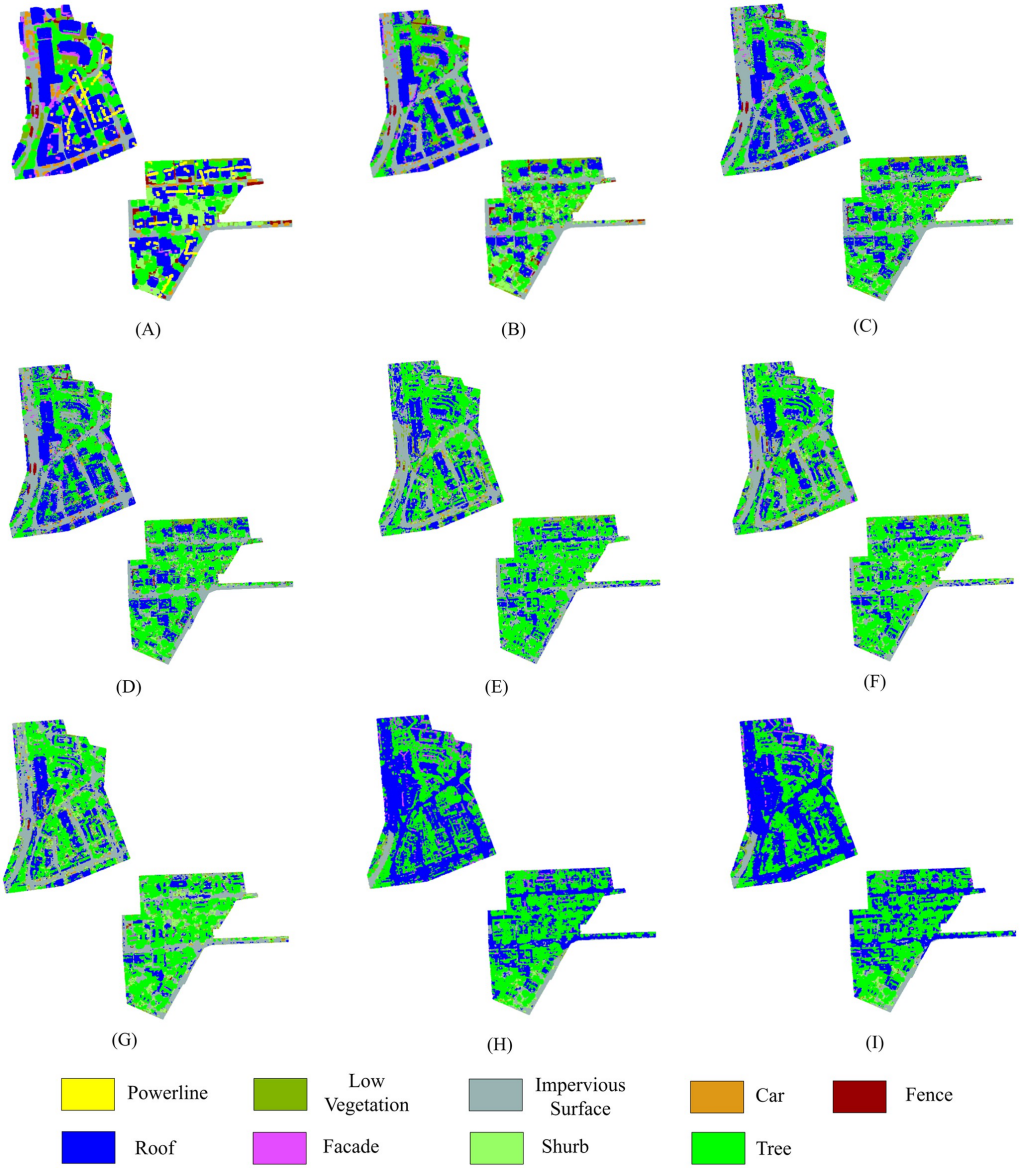
Visualization of segmentation outcomes for site 2 and site 3 within the Vaihingen dataset employing various neighborhood retrieval approaches. (A) Ground Truth, (B) Proposed Method, (C) Nong et al. (D) Xue et al. (E) He et al., (F) Günen, (G) Weinmann et al., (H) *k*=100, (I) *k*=50.

Fig 12B–12I shows the results of segmentation using different neighborhood selection methods along with two fixed-size neighborhoods for Vaihingen test areas. Fig 12A is the ground truth, and Fig 12B is the segmentation outcome of our proposed method, which clearly demonstrates that our proposed approach exhibits superior performance compared to other state-of-the-art methodologies.

**Fig 13:**
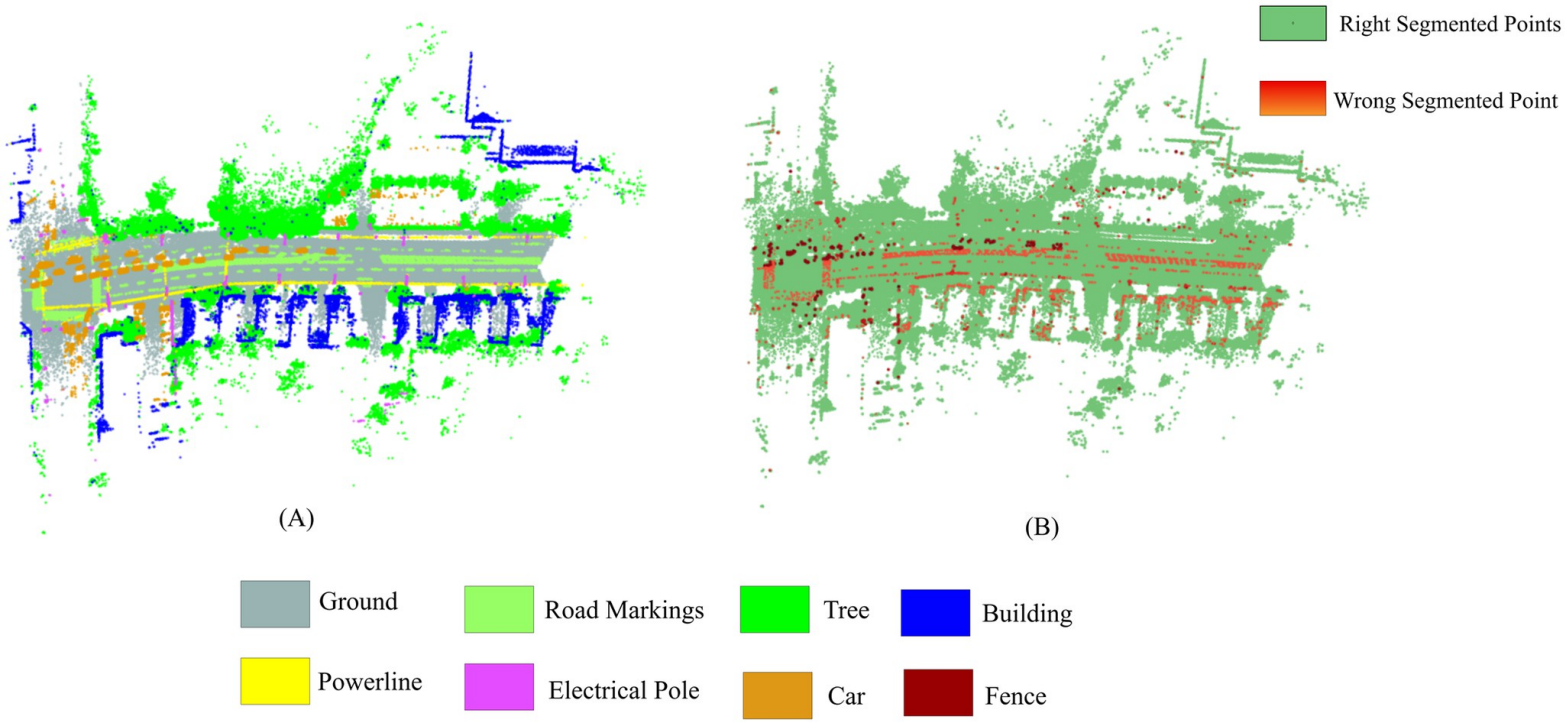
(A) Prediction map, and (B) error map of our proposed method on the Toronto-3D dataset test area.

**Fig 14:**
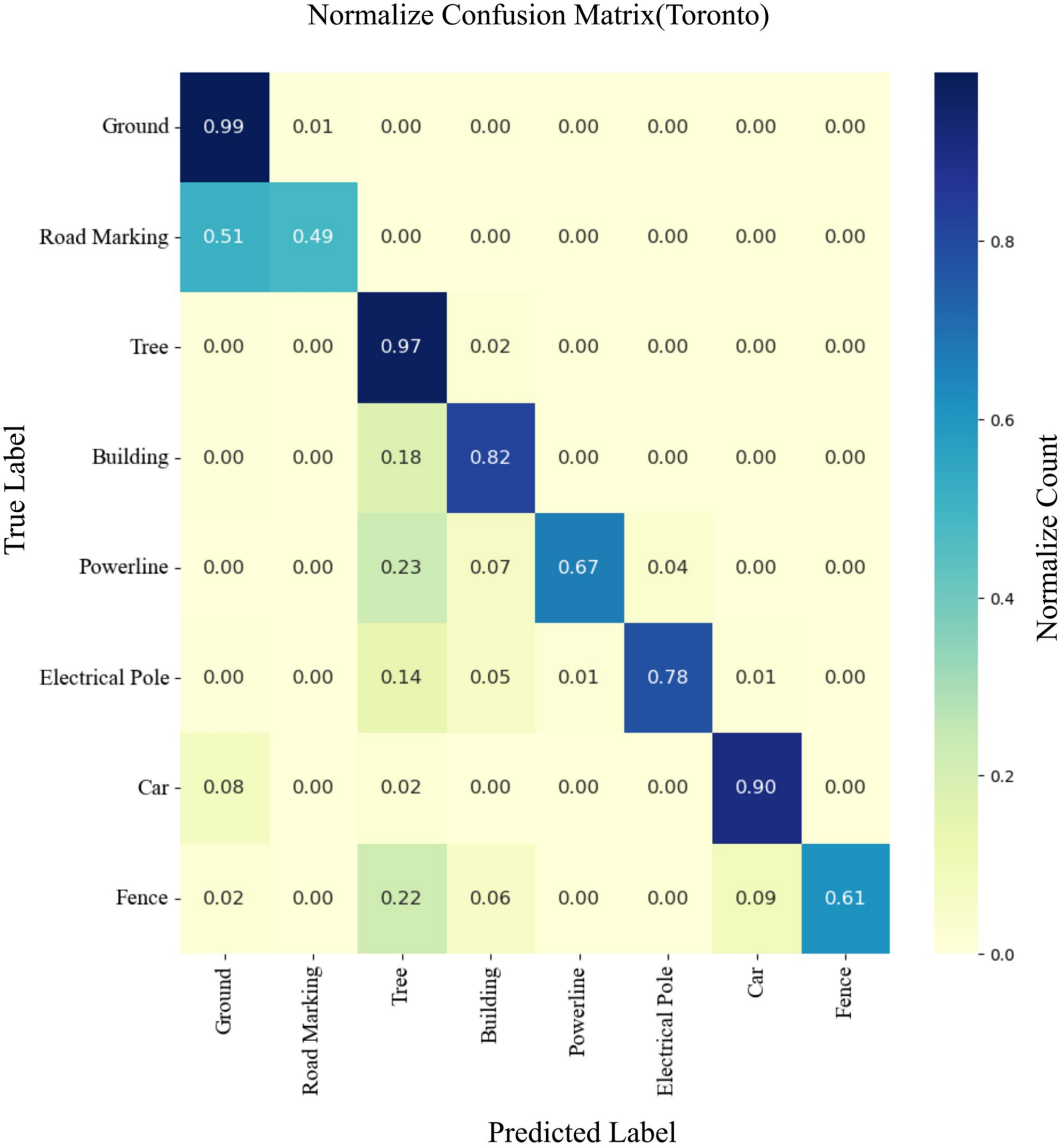
Confusion matrix of the machine learning classifier using the proposed approach on Toronto-3D dataset.

The efficiency of the proposed method is further validated through experimentation on an additional Toronto-3D dataset. The proposed approach generated promising results. To reduce computational effort, unclassified points in the Toronto dataset are not considered during training and testing. Fig 13A visually demonstrates the segmentation results by the created model on our test area, while Fig 13B shows an error map for misidentified points. Fig 14 depicts the confusion matrix of the machine learning model, encompassing the proposed neighborhood selection method, tested on the Toronto-3D dataset’s test area.

Table 6 presents a quantitative performance evaluation of the proposed neighborhood selection methods on the Toronto test dataset. The proposed approach is compared with the state-of-the-art methods of Sevgen et al. [60], Han et al. [61], and Huang et al. [62]. Specifically, our approach achieved an accuracy of 0.963, precision of 0.874, recall of 0.784, and F1-score of 0.825, which demonstrates the robustness and effectiveness of the proposed methodology.

**Table 6:** The accuracy, precision values, recall values, and F1-score values comparison according to different state-of-the-art methods of the Toronto-3D dataset.

Class	Accuracy	Precision	Recall	F1-score
Proposed	0.963	0.874	0.784	0.825
Sevgen et al. [60]	0.951	0.789	0.876	0.823
Han et al. [61]	0.936	-	-	-
Huang et al. [62]	0.812	-	-	0.451

## Discussion

Initially, we divided the input point cloud data into four regions by taking into account three distinct geometric natures: curvature, verticality, and omnivaraince. Neighborhoods should be selected based on the geometric nature of the input LiDAR point cloud data, and neighboring points must be chosen from similar regions. Considering this fact, we employed distinct adaptive neighborhood selection methods for different regions in our proposed approach. Furthermore, our machine learning-based classifier, which incorporates the proposed neighborhood approach based on selected features, outperformed current state-of-the-art deep learning and machine learning approaches, thereby improving urban scene segmentation results. The proposed approach yielded notably higher F1-score, precision, and overall accuracy compared to recent deep learning and machine learning approaches, as demonstrated in Tables 5 and 6. However, for some specific classes in both datasets, the proposed approach did not exhibit satisfactory segmentation performance. For instance, in the Vaihingen area, a significant number of points in the powerline and shrub classes were misidentified. This is primarily attributed to the sparse and imbalanced distribution of the input point cloud in the training dataset. Due to the same reasons, significant points in some classes in the Toronto3D dataset, including road markings and powerlines, are also misidentified.

## Conclusion

In this research, we have investigated the issue of 3D urban scene segmentation using LiDAR point cloud data. In the literature review section, we have pointed out that selecting an appropriate neighborhood for calculating the accurate feature value is the major issue in the existing approaches for segmenting the urban scene using machine learning classifiers. In this paper, we have proposed and used suitable neighborhood selection techniques based on different geometric properties of the individual points in different regions of the input LiDAR point cloud data. The experimental result section demonstrates the effectiveness of this research. We have used two different benchmark datasets to validate the impact of our method. The Vaihingen area of the ISPRS benchmark dataset has a low point density, and the Toronto3D dataset has a very high-density point cloud. In both cases, the proposed method demonstrates significantly high quantitative performance in terms of our selected evaluation metrics: Accuracy, Precision, Recall, and overall F1-score. We have also demonstrated significant qualitative performance in the experimental result section. However, the proposed method fails to show the expected segmentation performance for a few classes because of the comparatively fewer points in the training point cloud data and their confusing nature. We will specifically address this issue of enhancing the segmentation performance for those classes that have fewer points or imbalance classes with ambiguous characteristics in the future.
